# The Association between Social Vulnerability and Frailty in Community Dwelling Older People: A Systematic Review

**DOI:** 10.3390/geriatrics7050104

**Published:** 2022-09-26

**Authors:** Ayodele Ayeni, Adrienne Sharples, David Hewson

**Affiliations:** 1 Institute for Health Research, University of Bedfordshire, Luton LU1 3JU, UK; 2 Hertfordshire Partnership University National Health Service (NHS) Foundation Trust, Hatfield AL10 8YE, UK; 3 Faculty of Health and Social Sciences, School of Applied Social Science, University of Bedfordshire, Luton LU1 3JU, UK

**Keywords:** social vulnerability, frailty, older people

## Abstract

The aim of this systematic literature review was to determine whether social vulnerability is associated with frailty in older people. Databases were searched for literature from January 2001 to March 2022. Hand searches of reference lists of the selected articles were also used to identify other relevant studies. Studies that met the inclusion criteria were selected. Two independent reviewers assessed the methodological quality using an established tool. Eleven eligible studies from Canada, Europe, USA, Tanzania, Mexico, and China were selected. The level of social vulnerability measured by the Social Vulnerability Index (SVI) from a meta-analysis was 0.300 (95% CI: 0.242, 0.358), with the highest SVI in Tanzania (0.49), while the lowest level of SVI was reported in China (0.15). The highest frailty level of 0.32 was observed in both Tanzania and Europe, with the lowest frailty reported in a USA study from Hawaii (0.15). In all studies, social vulnerability was a significant predictor of mortality for both sexes at subsequent data collection points. The association between SVI and frailty was high in Tanzania (r = 0.81), with other studies reporting stronger correlations for females compared to males, but at small to moderate levels. In one study, an increase of 1SD in SVI was linked to a 20% increase in frailty score at a subsequent evaluation. Additional study is warranted to determine a potential causality between social vulnerability and frailty.

## 1. Introduction

The rise of the ageing population is considered one of the most rapidly changing demographic shifts, with the worldwide proportion of people aged over 60 forecast to more than double from 10% to 21% between 2000 and 2050 [1]. Generally, as age advances, individuals experience a decline in their social circumstances and become vulnerable to adverse health outcomes, which in turn pose pressures on social and healthcare services [2,3]. Research has shown that socially focused protective factors, such as social support, engagement in social networks, socioeconomic status and self-mastery, are associated with improved health status in older people [3].

Social vulnerability (SV) has emerged as a concept to holistically understand the range of social issues affecting the health of older people [4] and is regarded as the degree to which overall social situations render an individual susceptible to poor health outcomes. These adverse health outcomes are many and varied, and include prolonged hospitalization and institutionalization [3], an increase in pain [5], increased psychiatric consultations [6], mental health disorders [7] and falls [8]. Rather than focus on each individual factor for SV, a Social Vulnerability Index (SVI) has been developed that quantifies SV, with multiple facets of social circumstances considered [4].

Although SV is particularly important for individuals of all age groups, it is likely to have a greater impact in people aged 60 and above due to the increased incidence of significant social changes which can contribute to feelings of social isolation that people experience in later life [9]. These changes include retirement, death of a life partner, and dependency on others for self-care activities, such as shopping or personal hygiene [3,4]. For some older people, the accumulation of social changes can result in functional, psychological, and physiological decline [4], which are frequently recognised as components of frailty [10].

Frailty is regarded as a medical syndrome of high vulnerability to generalised decline and to a reduction in the ability to withstand commonly occurring everyday stressors [11]. Frail older people are more at risk for premature death and adverse health outcomes, including dementia, mobility decline and falls [12], institutionalization, hospitalization and increased health cost [12]. There are two major approaches that are commonly used to measure frailty. The first is the Frailty Phenotype (FP), which focuses on physical decline and is centred on the presence of three or more criteria of low grip strength, slow walking speed, exhaustion, reduced physical activities and unintentional weight loss [12]. The second approach is the Frailty Index, which is based on an accumulation of deficits model [10]. These deficits include signs, symptoms, disabilities, diseases. Social deficits are also included, many of which are also part of the SVI [10]. This includes several social deficits from the SVI that have been recognized as risk factors for the onset of cognitive decline [13]. Previous research has been able to identify social deficits as important factors implicated in the development of frailty and should be considered in long-term management of frailty. If SV was considered as a means of predicting the development of frailty [14] this construct could be a valuable method to determine when to intervene to prevent frailty in older people.

Although there has been an increase in the number of studies looking at the relationship between social vulnerability and frailty [3,7,15], there has yet to be any systematic review that synthesizes evidence from these studies on the association between social vulnerability and frailty. Hence, this present study aims at determining the prevalence of social vulnerability and frailty in older people, examining the relationship between social vulnerability and mortality, and finally exploring the association between social vulnerability and frailty in older people.

## 2. Materials and Methods

This systematic review followed the Preferred Reporting Items for Systematic Reviews and Meta-Analyses (PRISMA) guidance, following the PRISMA checklist [16] for systematic reviews and abstracts. The protocol for this systematic review was registered on Prospero (CRD42022306058). Ethical approval for the systematic review was obtained from the University of Bedfordshire Institute for Health Research Ethics Committee (IHREC922).

### 2.1. Search Strategy

The electronic databases searched were PsycINFO, SocINDEX, CINAHL, MEDLINE and Web of Science. Publication date was limited to dates between 2001 when the frailty phenotype study was first published [12] and March 2022. The search terms used were “Social vulnerab*” [Abstract/Title] AND Frail* [Abstract/Title]. In addition, MeSH terms for frailty were also used for the searches of MEDLINE and CINAHL databases. Hand searches of reference lists of the selected articles were used to identify other relevant studies.

### 2.2. Inclusion Criteria and Study Identification

Inclusion and exclusion criteria are shown in Table 1. These were based on the PICO acronym (Participants, Interventions, Comparisons and Outcomes) recommended by the Cochrane Library for systematic reviews [17]. Only studies published in English language were included in the review. The results of the search were imported into the EndNote reference management software (Version X9, Clarivate Analytics, Philadelphia, PA, USA) for deduplication and filtering. The articles retrieved were independently screened by AA and AS, with all articles selected by at least one reviewer included in a full-text review. Full-text review was carried out independently by the same two reviewers, with disagreements resolved by a third reviewer (DH).

### 2.3. Data Extraction and Management

Authors of eligible articles with missing data were contacted via email to retrieve relevant information. Information regarding social vulnerability and frailty were independently extracted from the eligible studies by AA and DH and compared, with no differences observed. Data extraction included all SVI and FI scores, including SVI scores for different frailty levels, where reported.

Meta-analysis was performed following the recommended methods of the Cochrane Handbook [18], with a random effects model used, with measures of I2 and Tau2 used to report levels of heterogeneity between studies [19]. Mean SVI scores were weighted across studies using the inverse variance for each study. Statistical significance was taken to be *p* < 0.05, with a Forest plot used to visualize the distribution of the SVI means from the different studies included. Meta-analysis was conducted using R-Studio [20], following the methods recommended in [21].

Correlations between social vulnerability and frailty were extracted from each article where they were reported, including correlations for sub-groups of the population, such as gender and age. The magnitude of correlations between social vulnerability and frailty was reported using Hopkins’ scale for effect size magnitude, from “extremely large” (r > 0.9), “very large” (0.7 ≤ r < 0.9), “large” (0.5 ≤ r < 0.7), “moderate” (0.3 ≤ r < 0.5), “small” (0.1 ≤ r < 0.3), down to “trivial” (r < 0.1) [22].

### 2.4. Quality Appraisal

The quality appraisal of the eligible articles was independently conducted by two authors AA and DH. The selected studies were appraised using the Downs and Black checklist [23], which contains 27 questions, of which 10 relate to reporting quality, three to external validity, while thirteen questions are on internal validity (bias and confounding) and one question on power. The checklist has been used widely because of its sub-scale divisions, which provides an opportunity to assess the profile of the weaknesses and the strengths of each methodological issue. Articles were rated on the scale of very good (26–28), good (20–25), fair (15–19), and poor (≤14) [24].

## 3. Results

### 3.1. Article Selection Process

**Figure 1:**
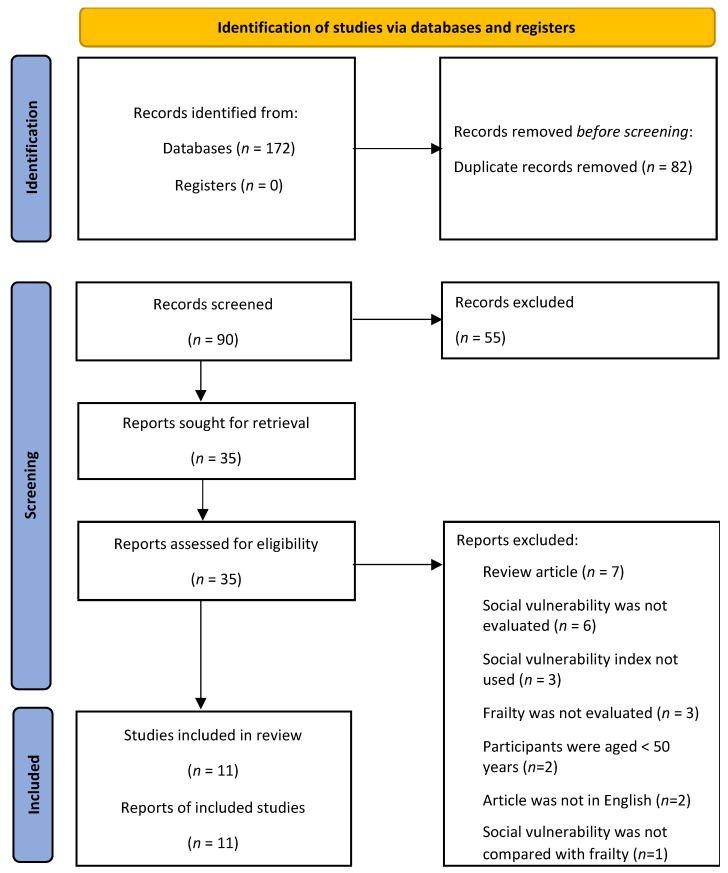
PRISMA flowchart of the systematic review.

The PRISMA flow chart [16] of the article selection process is shown in Figure 1. The preliminary search identified a total of 90 articles after duplicates were removed, of which 35 were retained for full-text screening. Twenty-one of these articles were rejected after full-text screening, with the remaining 14 selected articles shown in Table 2.

### 3.2. Study Characteristics

The selected studies were from Canada (4 studies), USA (3 studies), China, Mexico, and Tanzania (1 study each). Additionally, one study reported combined data for 12 European countries. Social vulnerability was assessed in all 11 studies using the SVI, which ranged from 18–49 items. Frailty assessment used the FI in all 11 studies, with a range of 31–60 items. Ten of the 11 included studies had quality appraisal scores rated as good, with one rated as fair [25].

### 3.3. Social Vulnerability and Frailty Levels

**Figure 2:**
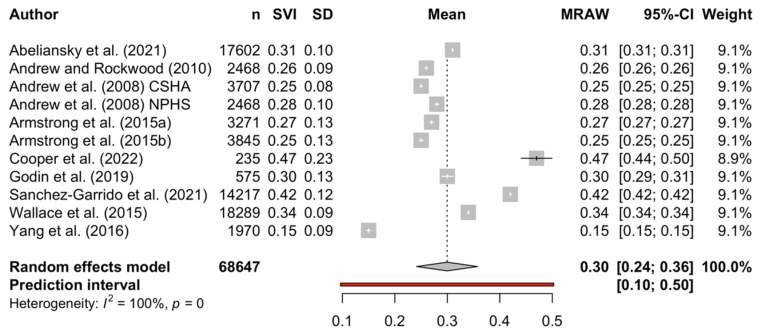
Forest plot of mean SVI [25,26,27,28,29,30,31,33,34,35].

The levels of social vulnerability and frailty are shown in Table 3. Studies reported social vulnerability in a variety of ways as either mean or median scores [25,26,27,28,29,30,31,32,33,34,35]. The mean/median of social vulnerability ranged from 0.15 in China [33] to 0.47 in Tanzania [35]. The meta-analysis for the single means produced a mean SVI of 0.300 (95% CI: 0.242, 0.358; *I*^2^ = 1.00, Tau^2^ = 0.01). A forest plot of the meta-analysis is shown in Figure 2. No studies reported the prevalence of social vulnerability since no accepted threshold has been defined for the SVI, however two studies classified participants as socially vulnerable using the highest quartile [30] and the upper tertile [34]. With respect to frailty, mean values reported for the FI ranged from 0.15 in Hawaii [27] to 0.32 in Europe [30] and in Tanzania [35].

### 3.4. Social Vulnerability and Mortality

The effect of social vulnerability on mortality in the cohort studies is shown in Table 4. In all studies, social vulnerability was a significant predictor of mortality at follow-up, ranging from 16 months to eight years [28]. When the effect of an increase in a single social deficit on mortality was reported, the odds ratios were 1.05 [28], 1.06 [35], and 1.08 [28] for the five-year, 16-month, and eight-year follow ups, respectively. Larger effects were observed when the highest quartile for SVI score was compared with the lowest quartile [30], or when the most socially vulnerable third of participants was compared to the least vulnerable third [32,34]. Finally, when a theoretical SVI of 1 was compared to an SVI of 0, the odds ratio for mortality was a large effect exceeding six [28].

### 3.5. Association between Social Vulnerability and Frailty

The relationship between social vulnerability and frailty was explored in five studies, all of which used SVI and FI, with mutually exclusive variables for both indexes in each study [25,26,28,33,35]. In Andrews’ original SVI study [28], small to moderate correlations between frailty and social vulnerability were reported, with results reported separately for men and women. With respect to men, correlations were r = 0.37 and r = 0.13 for the CSHA and NPHS cohorts, respectively [28]. In the same studies, stronger relationships were observed for women in both cohorts, with r = 0.47 and r = 0.24 for the CSHA and NPHS cohorts, respectively. In contrast, a stronger relationship was reported in the study in Tanzania (r = 0.81) [35]. In two studies [26,33], linear regression beta coefficients were reported for different frailty levels and social vulnerability levels. In the only longitudinal analysis, a 1 SD increase from the initial SVI resulted in a 20% increase in frailty at any age [25].

When SVI scores were compared between frailty levels, the frailest older people (FI > 0.3) had an SVI of 0.33 ± 0.14, compared to the fittest (FI < 0.1), who had an SVI of 0.25 ± 0.13 [26]. When different SVI levels were considered, lower social vulnerability buffered the effect of frailty on life satisfaction among younger participants when compared to those aged 80 years and above [33]. There was no relationship between study quality and any of the results found.

## 4. Discussion

This study investigated the relationship between social vulnerability and frailty by identifying the mean levels of each condition and examining the association between the two. Eleven selected studies from Canada, Europe, USA, Mexico, Tanzania, and China were eligible to be included in the review, with the level of social vulnerability varying between studies, and meta-analysis showing a mean SVI of 0.30. The highest SVI scores over 0.4 were reported in the Tanzania study in which 49 items were used [35], with the study from Mexico also reporting high social vulnerability [34]. These two studies used the greatest number of items to construct the SVI, which could be worthy of further study to identify whether a more comprehensive appraisal of SVI would result in higher levels of social vulnerability detected. Similar levels of around 0.3 were reported in Europe, irrespective of regional groupings [30] and North America [26,27,28,29,31,32]. In contrast, far lower levels were reported in China, where SVI levels were less than half those reported in Europe and North America [33]. This could be related to differences in social networks in China [36].

No studies reported the prevalence of social vulnerability, although two studies did classify the most vulnerable quarter [30] and third [34] of the population as highly socially vulnerable. It could be worthwhile to establish a threshold for SVI, similar to that used for FI, whereby anyone with an FI > 0.25 is considered to be frail [37]. This would enable SVI to be used at primary level for screening, similar to the way in which frailty screening is mandated in the NHS in England [38].

Five studies evaluated the relationship between social vulnerability and mortality [28,30,32,34,35]. In all cases, after adjusting for age, sex and frailty, social vulnerability had a significant effect on survival, with the effect increasing the greater the disparity between the levels of social vulnerability assessed. Such a finding shows that it could be worthwhile including social vulnerability assessment in health screening, as this could be an underlying factor in adverse health outcomes at a later stage in an individual’s life.

When the association between social vulnerability and frailty was evaluated in five studies [25,26,28,33,35], generally small to moderate correlations were reported, with stronger correlations observed in women than in men. However, in the study in Tanzania, a very strong association was observed, although this study had by far the highest level of social vulnerability [35]. This strong correlation could also be due to the subject selection process, which was weighted to include a higher proportion of frail and pre-frail participants. The differences between males and females could be due to the older age of the female participants in these studies, with age known to be a risk factor for increased frailty. Likewise, sex is also a risk factor for frailty, which could partly explain the relationship observed.

In those studies, where frailty groups were compared, there was an increase in social vulnerability in those with greater levels of frailty [33]. This review also found an interplay between frailty, social vulnerability, and life satisfaction. In the study in China, frail younger people who were socially vulnerable were likely to have low life satisfaction than old-old aged over 80 years old [33]. This could mean that being socially vulnerable when younger is more likely to have harmful effects than in older people.

In most studies, social vulnerability was considered as a single variable, however in one study there was an evaluation of different components of social vulnerability. Those factors that had the strongest relationship with frailty were social network size and loneliness in community dwelling older people.

This systematic review was the first to investigate whether social vulnerability was associated with frailty in community dwelling older people. Although several studies evaluated the relationship between social vulnerability and frailty, most of these reported only cross-sectional results, other than the relationship between social vulnerability and mortality. However, in the only study in which a longitudinal analysis of social vulnerability and frailty was reported, increased social vulnerability at the follow-up evaluation was linked to an increase in frailty [25].

The strengths of this review include the rigorous and extensive systematic literature search for identifying eligible articles on the narrow subject of the relationship between SVI and frailty in older adults. The review identified differences in SVI levels between countries, however none of the studies considered differences in ethnicity or migrant status when comparing SV and frailty, despite previous studies highlighting the importance of these factors in frail older people [39].

The major limitation of this study is that there were limited articles exploring the relationship between SV and frailty in older people. In addition, SV and frailty were inconsistently assessed across studies with the use of a variety of measuring tools for both conditions, making it difficult to draw any firm conclusions. Finally, only one study included in the review explored any causal relationship between the conditions.

## 5. Conclusions

This systematic review demonstrated that different levels of both SV and frailty exist between countries, with generally low to moderate correlations identified between social vulnerability and frailty. However, additional study is warranted to determine a potential causality between social vulnerability and frailty.

## Figures and Tables

**Table 1:** Inclusion and exclusion criteria based on PICO methodology.

Terms	Inclusion Criteria	Exclusion Criteria
P Population	Community-dwelling older people Aged 50 and above	Studies involving populations in care homes Population included people aged below 50 years
I Intervention/exposure	Social vulnerability measured using a social vulnerability index	Social vulnerability measured using another tool
C Comparison	None	None
O Outcome	Frailty measured using a recognized tool	No record of frailty as the outcome or not measured with a recognized tool
S Study design	Cohort studies, cross-sectional studies	Literature review or systematic review

**Table 2:** Characteristics of the selected articles.

Authors	Participants	Sample (*n*, Age)	Gender (% Female)	Study Design	SVI Items	FI Items	Quality Appraisal
Abeliansky et al. (2021)	USA HRS	17,602 Aged 50+ 68.3 ± 9.7	57%	Cohort 12 years	49	38	18
Andrew et al. (2008)	Canada CSHA	3707 77.9 (77.8, 78.1)	60%	Cohort 5 years	40	31	24
	Canada NPHS	2468 73.4 (73.0, 73.7)	58%	Cohort 8 years	23	36	22
Andrew and Rockwood (2010)	Canada CSHA	2468 Age 70+	NS	Cohort 5 years	40	31	23
Andrew et al. (2012)	Canada CSHA-2	5703 Age 70+	NS	Cohort 10 years	40	31	22
Armstrong et al. (2015a)	Hawaii, USA HAAS	3271 Aged 72+	Men only	Cohort 3–6 years	19	58	22
Armstrong et al. (2015b)	Hawaii, USA HAAS	3845 Aged 71+	Men only	Cohort 3–6 years	18	48	22
Cooper et al. (2022)	Tanzania	235 Aged 60+ 75.2 ± 11.5	58%	Cohort 13–19 months	45	37	21
Godin et al. (2019)	Canada	475 Aged 65+ 78.6 ± 7.9	58.9%	Cross- sectional	18	39	21
Sanchez-Garrido et al. (2021)	Mexico MHAS	14,217 Age 50+ 63.9 ± 10.1	59%	Cohort 3 years	42	60	20
Wallace et al. (2015)	Europe SHARE	18,289 Age 50+	NS	Cohort 5 years	32	57	22
Yang et al. (2016)	China	1970 Age 65+	NS	Cross- sectional	35	52	21

CSHA: Canadian Study of Health and Aging; FI: Frailty Index; HAAS: Honolulu-Asia Aging Study; HRS: Health and Retirement Study; MHAS: Mexican Health and Aging Study (MHAS)NPHS: National Population Health Survey; SHARE: Survey of Health, Ageing and Retirement in Europe; NS: not specified; SVI and FI values are the number of items used in each scale. Ages expressed in years.

**Table 3:** Levels of social vulnerability and frailty.

Authors	Country	Social Vulnerability	Frailty
Abeliansky et al. (2021)	USA	0.31 ± 0.10	0.23 ± 0.16
Andrew et al. (2008)	Canada	CSHA: 0.25 (0.20, 0.31) ‡	Only histograms provided
		NPHS: 0.28 (0.21, 0.35) ‡	
Andrew and Rockwood (2010)	Canada	0.26 ± 0.09	0.16 (0.10, 0.23) ‡
Andrew et al. (2012)	Canada	Not reported	Not reported
Armstrong et al. (2015a)	Hawaii, USA	0.27 ± 0.13 §	0.15 ± 0.08 §
Armstrong et al. (2015b)	Hawaii, USA	0.25 ± 0.13 §	0.15 ± 0.09 §
Cooper et al. (2022)	Tanzania	0.47 (0.23)	0.32 (0.35)
Godin et al. (2019)	Canada	0.30 ± 0.13 §	0.19 ± 0.10 §
Sanchez-Garrido et al. (2021)	Mexico	0.42 ± 0.12 §	0.23 ± 0.11 §
Wallace et al. (2015)	Europe	Total 0.34 ± 0.09 § Nordic 0.31 ± 0.86 § Continental 0.33 ± 0.09 § Mediterranean 0.36 ± 0.09 §	Total 0.316 ± 0.12 § Nordic 0.13 ± 0.11 § Continental 0.16 ± 0.12 § Mediterranean 0.18 ± 0.13 §
Yang et al. (2016)	China	Total 0.15 ± 0.09 § 65–79 0.14 ± 0.09 § 80+ 0.18 ± 0.09 §	Total 0.21 ± 0.15 § 65–79 0.18 ± 0.12 § 80+ 0.30 ± 0.18 §

§ Data are means ± SD; ‡ Data are medians and inter-quartile ranges.

**Table 4:** Effect of social vulnerability on mortality.

Authors	Dataset	Follow Up	Comparison	Adjustments	*n*	Effect Statistic (95% CI)
Andrew et al. (2008)	CSHA	5 years	One more deficit	Age, sex, frailty	3707	1.05 (1.02, 1.07) §
						1.03 (1.01, 1.05)
	NPHS	8 years	One more deficit	Age, sex, frailty	2468	1.08 (1.03, 1.14) §
						1.04 (1.01, 1.07)
Andrew et al. (2012)	CSHA	5 years	Highest third of SVI compared to lowest third	Age, sex	584	2.5 (1.5, 4.3)
				Age, sex, physical activity, smoking, alcohol		3.22 (1.80, 5.78)
Cooper et al. (2022)	N/A	16 months	One more deficit	Age, gender, frailty	235	1.06 (0.97, 1.16)
Sanchez-Garrido et al. (2021)	MHAS	3 years	Highest third of SVI compared to lowest third	Physical activity, tobacco, alcohol	14,217	1.7 (1.2, 2.3)
Wallace et al. (2015)	SHARE	5 years	Highest quartile of SVI	Age, sex	18,289	1.88 (1.64, 2.16)
				Age, sex, frailty, disability	18,289	1.25 (1.07, 1.45)

Effect statistics are hazard ratios unless otherwise indicated; Effect statistics are odds ratios §.

## Data Availability

Not applicable.
